# MRI-Based Machine Learning and Radiomics Methods for Assessing Spinal Cord Function in Patients with Mild Cervical Spondylotic Myelopathy

**DOI:** 10.3390/bioengineering12060666

**Published:** 2025-06-17

**Authors:** He Wang, Kai Wang, Yutian Wang, Zhenlei Liu, Lei Zhang, Shanhang Jia, Kun He, Xiangyu Zhang, Hao Wu

**Affiliations:** 1 Department of Neurosurgery, Xuanwu Hospital, Capital Medical University, 45 Changchun St, Xicheng District, Beijing 100053, China; wanghe_15@126.com (H.W.); wangkaispine@xwh.ccmu.edu.cn (K.W.); wangyutian@mail.ccmu.edu.cn (Y.W.); zhenlei.liu@xwhosp.org (Z.L.); zhanglei@xwhosp.org (L.Z.); shanhangjia@126.com (S.J.); zhangxiangyu@mail.ccmu.edu.cn (X.Z.); 2 Department of Neurosurgery, China-Japan Friendship School of Clinical Medicine, Peking University, No. 2 Yinghuayuan East Street, Chaoyang District, Beijing 100029, China; hekun39@foxmail.com; 3 Department of Neurosurgery, China-Japan Friendship Hospital, No. 2 Yinghuayuan East Street, Chaoyang District, Beijing 100029, China

**Keywords:** cervical spondylotic myelopathy, PET-MRI, radiomics, machine learning

## Abstract

(1) Background: Patients with mild cervical spondylotic myelopathy (CSM) who delay surgery risk progression. While PET evaluates spinal cord function, its cost and radiation limit its use. (2) Methods: In this prospective study, patients with mild cervical spondylosis underwent preoperative 18F-FDG PET-MRI. Narrowed spinal levels were classified based on whether SUV_max_ was decreased. Follow-up assessments were conducted. Two machine learning models using MRI T2-based radiomics were developed to identify stenotic levels and decreased SUV_max_. (3) Results: Patients with normal SUV_max_ showed greater symptom improvement. The radiomics models performed well, with AUCs of 0.981/0.962 (training/testing) for stenosis detection and 0.830/0.812 for predicting SUV_max_ decline. The model outperformed clinicians in predicting SUV_max_ decline, improving the AUC by 10%. (4) Conclusion: Patients with preserved SUV_max_ have better outcomes. MRI-based radiomics shows potential for identifying stenosis and predicting spinal cord function changes for preoperative assessment, though larger studies are needed to validate its clinical utility.

## 1. Introduction

Degenerative cervical myelopathy (DCM) is the most common cause of spinal cord dysfunction and most importantly of patients with mild myelopathic symptoms [1]. According to AOSpine clinical practice guidelines for the management of DCM, the quality of evidence is low for providing surgical intervention for mild cervical spondylotic myelopathy (CSM) patients [2]. Mild CSM patients may initially refuse surgery due to mild symptoms; however, a significant proportion (20–62%) experience worsening conditions within 3–6 years, necessitating surgical intervention [3,4]. Therefore, the timely assessment of spinal cord function in mild CSM patients is crucial for determining surgical intervention and preventing disease progression.

Positron Emission Tomography (PET) based on 18F-FDG is currently an important method for evaluating cervical spinal cord function [5,6,7,8]. Previous studies have indicated that cervical spondylosis patients can be classified into two types based on the maximum standardized uptake value (SUV_max_): Type 1, with an increased or normal SUV_max_ in the compressed segment, characterized by a short medical history and rapid disease progression; and Type 2, with a decreased SUV_max_ in the compressed segment, characterized by a long medical history and chronic disease progression [9]. Previous research showed that patients with Type 1 cervical spondylosis typically experience significant improvement in symptoms after surgery, while symptoms in Type 2 patients show less noticeable improvement [10]. Therefore, conducting PET scans on patients can preoperatively assess spinal cord function, which is of significant importance for predicting patient prognosis. However, there is currently no research describing whether PET has evaluative value for spinal cord function in mild CSM patients. Furthermore, due to the radioactive nature of PET and its relatively high cost, conducting PET scans for all CSM patients is not advisable. Consequently, there is an urgent need for an alternative to PET for assessing spinal cord function.

To reduce radiation exposure and hospitalization costs, utilizing MRI to assess spinal cord function has become a main alternative to PET. Some studies reported a negative correlation between the high abnormal intramedullary signal on preoperative T2-weighted images and neurological improvement rates [11]. However, one study indicated that the signal on T2-weighted images does not correlate with prognosis [12]. Therefore, there remain challenges to objectively assess cervical spinal cord function using MRI.

Radiomics is a method of extracting numerous features from images and creating models based on these features to accurately analyze images [13]. Currently, numerous studies have employed radiomics to extract imaging features such as intracranial lesions and pathological changes, modeling predictions for aspects like drug treatment response and prognosis [14,15]. However, there is still a lack of reported research on spinal cord function-related studies based on radiomics.

In this study, we constructed a model based on radiomics to assess spinal cord function. The main contributions are as follows:

1. We prospectively collected a cohort of mild CSM patients and elucidated the relationship between decreased SUV_max_ and prognosis.
2. We constructed a model based on radiomics capable of identifying compressed cervical segments.
3. We developed a model based on radiomics capable of identifying segments with decreased SUV_max_.
4. We conducted feature analysis on the models, yielding radiomic indicators with clinical relevance to guide clinical practice.

## 2. Methods

### 2.1. Study Population

This study prospectively enrolled mild CSM patients at our hospital, from January 2023 to June 2023. Although, according to AOSpine clinical practice guidelines, mild cervical spondylotic myelopathy (CSM) is defined by modified Japanese Orthopaedic Association (mJOA [16]) in the range of 15–17, to enroll more patients, we selected relatively mild CSM patients with mJOA ≥ 14 [2].

Inclusion criteria were (1) a radiological diagnosis of cervical spinal cord compression, (2) preoperative PET and MRI imaging examinations, and (3) relatively mild symptoms (mJOA ≥ 14). Exclusion criteria were (1) cervical spine tumors, (2) tuberculosis or other infectious diseases, and (3) a history of previous cervical spine surgery.

All patients were reassessed for mJOA scores 6 months after surgery, and the improvement in mJOA scores was calculated to evaluate treatment efficacy.

### 2.2. Image Scanning Process and Analysis

For all patients, preoperative PET-MRI scans were conducted using a scanner from UTH Manufacturer, with MRI sequences including coronal, sagittal, and axial T1- and T2-weighted imaging. The T2-weighted coronal parameters used for analysis were TR 4690 ms, TE 114 ms, pixel spacing of 0.4584 mm, and slice thickness of 3.3 mm. All patients underwent a minimum 12 h fasting period before PET studies. A dose of 370 MBq of 18F-FDG, with an effective radiation dose of 10 mSv, was injected one hour before the PET studies. The scanned PET images were limited to the cervical region, with a pixel spacing of 3.125 mm and a slice thickness of 2.78 mm.

The 3D-Slicer software (version 5.3.0) was employed to annotate the spinal cord in the five intervertebral disc segments (C2/3 to C6/7) by two neurosurgeons with 5 years of experience. The SUV_max_ of PET within these five segmental regions of interest (ROIs) was calculated [17]. These values represented the functional status of each cervical spinal cord segment.

### 2.3. Radiomic Feature Extraction

Based on segmented images, features were extracted from the axial T2-weighted imaging sequences using the pyradiomics software package (version 3.0) (https://github.com/Radiomics/pyradiomics, accessed on 1 January 2024). Due to the relatively small spinal cord area, a small sampling preprocessing step was designed to achieve finer resolution. Firstly, all images were resampled to isotropic voxels of 0.5 × 0.5 × 0.5 mm^3^. Voxel intensities were normalized and discretized at intervals of 5. Subsequently, four Laplacian of Gaussian (LoG) filters with sigma values ranging from 0.5 to 2.0 at intervals of 0.5 were used. Finally, 1197 radiomic features were extracted.

### 2.4. Clinical Task Setting

In this study, two tasks were defined for spinal cord segments. The first task involved determining whether the spinal cord segment was compressed, with two spinal cord imaging experts with over 10 years of experience making decisions to assess the degree of compression in each segment. The second task was to assess the function of the spinal cord segment, based on the SUV_max_ values from PET scans. If the SUV_max_ value in a level decreased by 10% compared to the nearest normal level, it indicated impaired spinal cord function. Segments with increased or unchanged SUV_max_ were considered to have intact spinal cord function.

Therefore, please note that in this study, levels with normal or increased SUV_max_ were both listed as normal SUV_max_ levels.

The training and testing sets were randomly divided in a 3:1 ratio at the patient level, with all five cervical spinal cord segments (C2/3 to C6/7) extracted for validation in task one (determining whether the cervical spinal cord is compressed). For task two (determining whether the cervical spinal cord function is impaired), only compressed segments were used for training and validation.

### 2.5. Construction of the Machine Learning Model

For both clinical tasks, the same procedure was employed to construct machine learning models. Over 1197 clinical features were extracted using the feature extraction method. To eliminate irrelevant and redundant data, intraclass correlation coefficient (ICC) values and hierarchical clustering were used to select features in an unsupervised learning manner [13]. To further select representative features, recursive feature elimination (RFE) was employed for supervised feature selection. Nine typical machine learning algorithms, including LinearSVC, Random Forest Classifier [18], Extra Trees [19], KNN, Decision Trees [20], Gbdt [21], AdaBoost [22], MLP [23], and XGBoost [24], were chosen for model construction. The area under the curve (AUC) was used as the final evaluation metric. To objectively assess model quality, accuracy, sensitivity, specificity, PPV, and NPV indices were also calculated for each model.

Initially, the nine typical machine learning models were built based on the training set. Five-fold cross-validation in the training dataset was used to reduce overfitting and validate the robustness of the results. Subsequently, the trained models were further validated on the test set. The judgments of three clinical experts, including two with 3 years of spinal cord imaging experience and one with 10 years of experience, were compared with the constructed models in the test set.

### 2.6. Model Interpretation

Machine learning models with a large number of parameters perform well but lack interpretability, resembling a black box. To track the contribution of each input feature in the machine learning model, we introduced an explanation technique called Shapley Additive exPlanations (SHAPLEY) [25]. This technique calculates the marginal contribution when adding a feature to the model. In SHAP, the attributes of each feature (protective or risk factor) are thoroughly explained through marginal contributions.

For individual interpretation, we introduced the Local Interpretable Model-Agnostic Explanations (LIME) algorithm, which approximates the model’s expression and explains the contribution of each feature to a specific patient [26].

### 2.7. Statistical Analysis

AUC calculations were performed using IBM SPSS Statistics 23.0 software (IBM Corporation). Image annotations were conducted using 3D-Slicer. Data processing was carried out with Python 3.7, and the models were constructed using the sklearn package. A two-sided *p*-value less than 0.05 was considered statistically significant.

## 3. Results

### 3.1. Patient Demographics

A total of 24 patients from our hospital were included in this study. Based on the SUV_max_, patients were divided into two groups, with 10 patients having decreased SUV_max_ segments and 14 patients having normal (not decreased) SUV_max_ segments. There were no significant differences in age, gender, BMI, and disease course between the two groups (Table 1). Preoperative mJOA scores and follow-up mJOA scores at postoperative six months also showed no significant differences. However, in terms of mJOA improvement, the group with normal SUV_max_ exhibited a slightly greater improvement (*p* = 0.043).

Furthermore, the 24 patients were divided into a training set (n = 18) and a validation set (n = 6), totaling 116 segments. In task one, 18 patients with 87 segments were enrolled in the training set, while 6 patients with 29 segments were enrolled in the test set. As described in Table 2, among the included patients, there were no compressed segments in the C2/3 level. The most commonly compressed segment was C5/6 (87.5%), followed by C4/5 (75%). In task two, a total of 56 segments were included, with 42 segments as the training set and 14 segments as the test set. Table 3 shows that the segments most prone to a decrease in PET uptake were C5/6 (42.86%) and C6/7 (40%). This suggests that the lower cervical spinal cord is more likely to experience a decrease in uptake when compressed, indicating a poorer prognosis.

### 3.2. Machine Learning Assessment of Spinal Cord Compression

**Figure 1:**
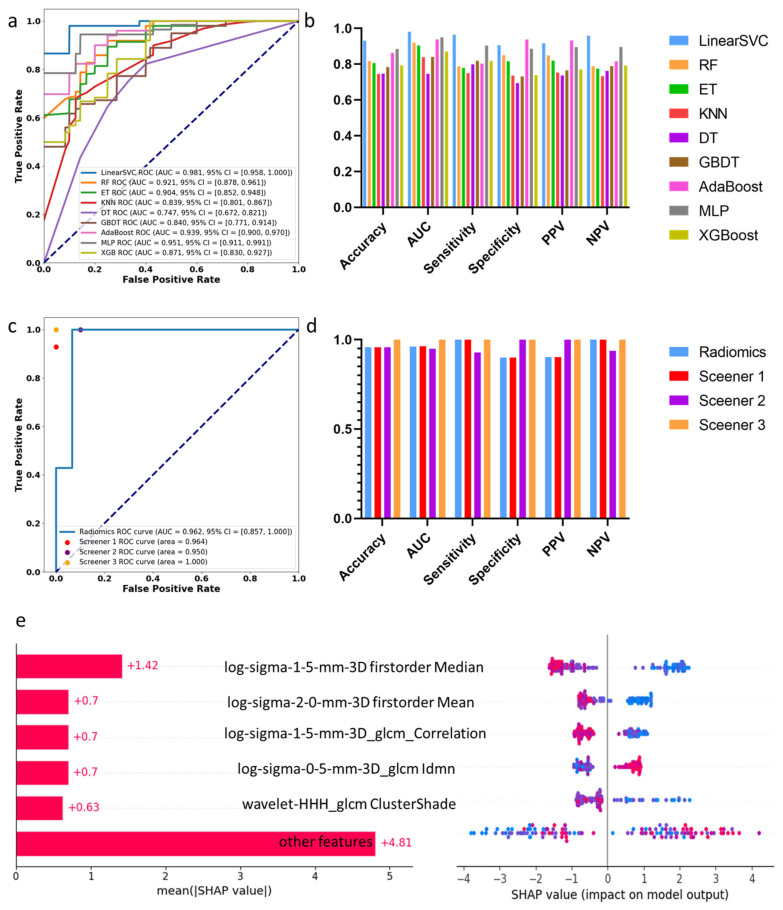
Results of identifying compression levels: (**a**) AUC curve of 9 machine learning algorithms on the training dataset; (**b**) statistic comparison of 9 machine learning algorithms on the training dataset; (**c**) AUC curve of proposed machine learning algorithm and 3 screeners on the test dataset; (**d**) statistic comparison of proposed machine learning algorithm and 3 screeners on the test dataset; (**e**) SHAPLEY explanations of the proposed model.

Figure 1 shows the results of identifying compressed segments. After training and five-fold cross-validation, the LinearSVC model performed the best with an AUC of 0.981 in the training dataset. It achieved an accuracy of 0.93, sensitivity of 0.96, and specificity of 0.91. In the test set, the radiomics model obtained an AUC of 0.962 and an accuracy of 0.96. The AUC values from three clinical experts were 0.964, 0.950, and 1, with accuracies of 0.96, 0.96, and 1, respectively. These data suggest that the physicians involved were skilled at identifying spinal cord compression, and the machine learning models did not significantly outperform their assessments (*p* = 0.423).

Through SHAPLEY feature analysis (Figure 1e), we found that the T2 low signal (radiomics feature: firstorder Median, which represents the median signal of the ROI) in radiomics is a prominent parameter in determining compression. However, we considered that the exclusion of disc compression and surrounding cerebrospinal fluid in radiomics is a limitation resulting in low accuracy, as cerebrospinal fluid signal around the spinal cord is essential for clinical assessments. The focus on only the ROI rather than the entire image might be one reason why machine learning algorithms do not outperform clinical experts. Additionally, factors such as limited sample size and variability in physician expertise may also influence the outcomes.

### 3.3. Machine Learning Assessment of Segments with Decreased SUV_max_

**Figure 2:**
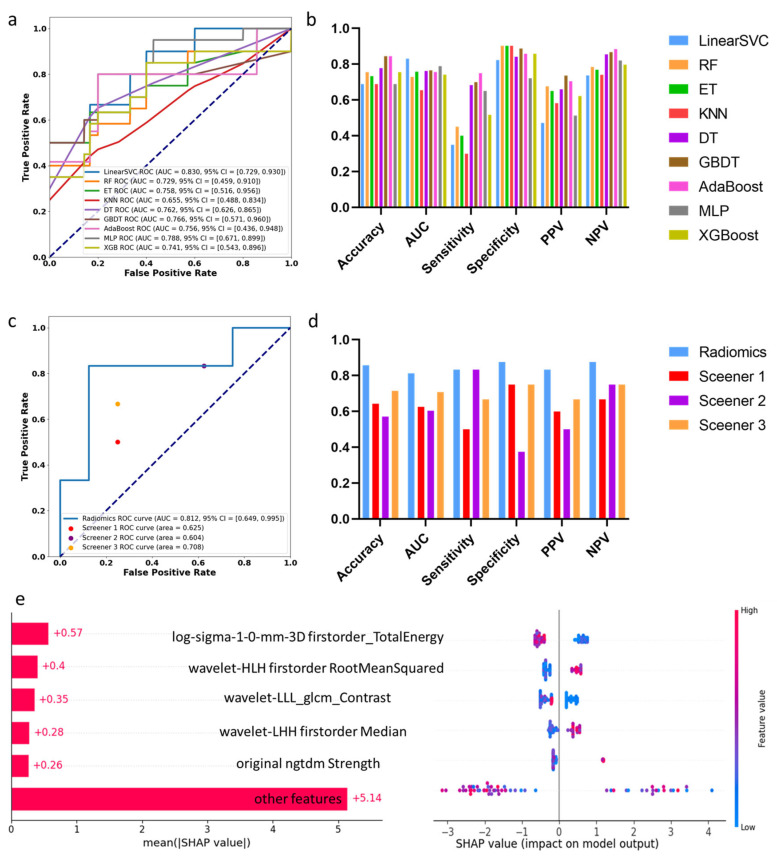
Results of identifying decreased ^18^F-FDG uptake levels: (**a**) AUC curve of 9 machine learning algorithms on the training dataset; (**b**) statistic comparison of 9 machine learning algorithms on the training dataset; (**c**) AUC curve of proposed machine learning algorithm and 3 screeners on the test dataset; (**d**) statistic comparison of proposed machine learning algorithm and 3 screeners on the test dataset; (**e**) SHAPLEY explanations of the proposed model.

Figure 2 shows the results of identifying decreased 18FDG uptake segments. Based on training and five-fold cross-validation results, the LinearSVC model performed the best with an AUC of 0.830 and an accuracy of 0.69. In the test set, the radiomics model obtained an AUC of 0.812 and an accuracy of 0.86. The AUC values from three clinical experts were 0.625, 0.604, and 0.708, with accuracies of 0.64, 0.57, and 0.71. The radiomics-based machine learning model significantly outperformed clinical doctors in detecting segments with decreased SUV_max_ with higher accuracy (*p* = 0.035).

SHAPLEY feature analysis revealed that TotalEnergy, related to area and signal, had a high contribution. TotalEnergy indicated that segments with decreased SUV_max_ had a smaller cross-sectional area, signifying more severe compression. RootMeanSquared, associated with signal disorder within the ROI, indicated that levels with decreased SUV_max_ had more chaotic T2 signals. Additionally, a higher median T2 signal value was significantly correlated with decreased SUV_max_. Radiomics-based machine learning, focusing on ROI’s abnormal signals, far surpassed clinical doctors in accuracy, with AUC values exceeding 0.8 in both training and test sets, indicating the potential to assess spinal cord function based on T2-weighted imaging.

### 3.4. Individual Feature Interpretation Based on LIME

To better understand the reasons behind predicting decreased SUV_max_, LIME was employed for feature interpretation of the constructed model.

**Figure 3:**
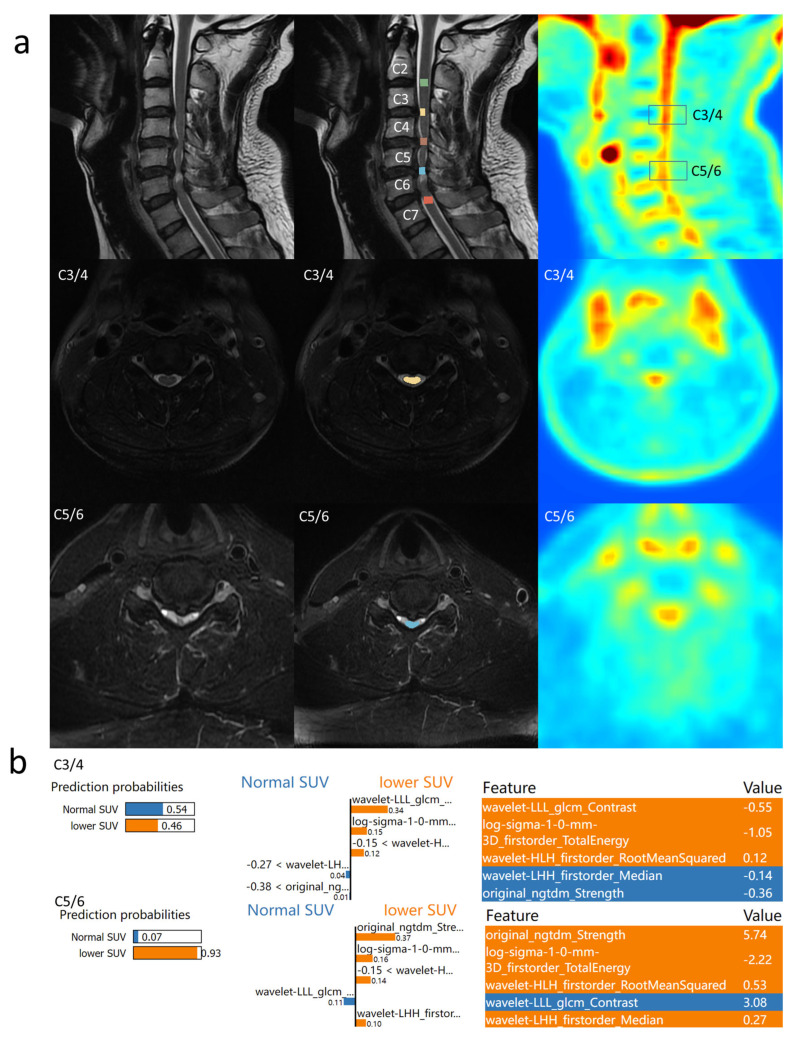
Prediction result of Example 1: (**a**) the PET/MR results of Example 1 are shown. Cervical spinal cord segments from C2 to C7 and five intervertebral disc segments were labeled. SUV_max_ was normal at the C3/4 level and SUV_max_ was decreased at the C5/6 level; (**b**) the LIME results of the proposed model are shown.

Example 1 (Figure 3): A patient with cervical compression in C3 to C7 segments, where C3/4 showed unchanged 18FDG uptake, but C4/5, C5/6, and C6/7 exhibited decreased 18FDG uptake. The model indicated a 0.54 probability of normal SUV_max_ in C3/4, primarily due to its lack of significant signal elevation (Median = −0.14). However, other features leaned towards predicting decreased uptake, resulting in a more neutral probability. In C5/6, the model predicted a 0.93 probability of decreased SUV_max_, associated with small TotalEnergy, chaotic signals, and a higher median T2 signal.

**Figure 4:**
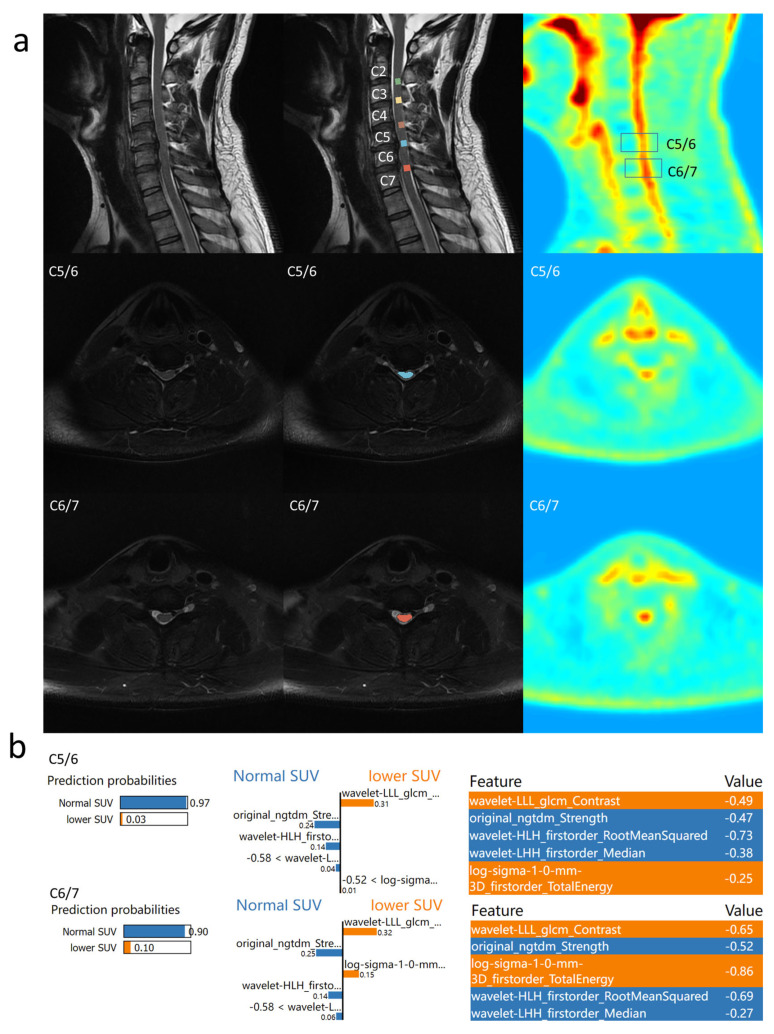
Prediction result of Example 2: (**a**) the PET/MR results of Example 2 are shown. Cervical spinal cord segments from C2 to C7 and five intervertebral disc segments were labeled. SUV_max_ decreased at the C5/6 level and SUV_max_ was normal at the C6/7 level; (**b**) the LIME results of the proposed model are shown. The proposed methods predicted that both SUV_max_ were normal at the two levels.

Example 2 (Figure 4): Another patient with compression in C5/6 and C6/7, where C5/6 showed decreased SUV_max_, but C6/7 had a normal SUV_max_. In C5/6, the model predicted a 0.97 probability of normal SUV_max_, considering the absence of high median signals and non-chaotic signals. In C6/7, the model predicted a 0.90 probability of normal SUV_max_ due to the absence of high median signals and non-chaotic signals, resulting in successful prediction. This indicates that although the selected features are representative, they are not sufficient to fully replace PET in reflecting cervical cord function.

## 4. Discussion

In this study, a prospective collection of a cohort of mild CSM patients was conducted, followed by the development of a machine learning algorithm based on radiomics to automatically identify compressed segments and predict SUV_max_ reduction in the compressed segments. These two tasks achieved AUC values of 0.981 and 0.830 in the training set and 0.962 and 0.812 in the test set, respectively.

### 4.1. Deep Learning and Machine Learning Applications for CSM Patients

For the automatic identification of compressed segments, a review indicates that the diagnostic accuracy of disc herniation or spinal stenosis based on deep learning is already above 0.95, similar to clinical doctors’ accuracy [27]. However, diagnostic performance varies across imaging modalities. Deep learning models developed for degenerative cervical myelopathy have demonstrated superior inter-rater agreement in diagnosing spinal canal stenosis on MRI (κ = 0.78, *p* < 0.001 vs. κ range = 0.57–0.70 for human readers) and classifying foraminal stenosis (κ = 0.80, *p* < 0.001 vs. κ range = 0.63–0.69), with diagnostic accuracy comparable to manual interpretation (DL: 92.3%, readers: 92.3–100.0%) [28]. In contrast, deep learning approaches based on X-ray imaging have shown significantly improved accuracy, far surpassing human interpretation (89.7% vs. 68.3%) [29].

Regarding prognosis, machine learning models have achieved an AUC of 0.777 in predicting the length of hospital stay following cervical spine surgery [30]. Another study reported that machine learning models attained average AUC values of 0.776, 0.846, 0.775, and 0.747, predicting a prolonged length of stay, non-home discharges, 30-day remissions, and major complications, respectively [31].

Deep learning in image recognition comprehensively understands semantic information throughout the entire image, whereas radiomics-based machine learning understands only the texture features in the annotated ROI [32]. In the task of identifying spinal cord compression, changes in morphological structure are crucial, and radiomics cannot integrate this feature. SHAPLEY analysis revealed that spinal cord compression not only involves morphological changes but also often includes a reduction in the intramedullary T2 signal in the compressed segment.

### 4.2. Association Between PET/MRI and Prognosis

Current research indicates that PET is a crucial examination for assessing spinal cord functional reserve and predicting prognosis [33]. Frank’s studies demonstrate a significant correlation between SUV_max_ values in PET and prognosis [10]. Segments with increased SUV_max_ values show significant postoperative symptom improvement (*p* = 0.001), while those with decreased SUV_max_ values show no significant improvement postoperatively (*p* > 0.05). Similarly, Kenzo found a high correlation between SUV ratio (SUVR) and neurological improvement (R = 0.837, *p* = 0.001) [12]. Therefore, predicting whether SUV_max_ decreases has significant implications for prognosis, indirectly representing spinal cord function. In our prospective cohort study, we similarly found more significant symptom improvement in the normal SUV_max_ group (*p* = 0.043). Additionally, we observed that the lower cervical cord is prone to decreased SUV_max_ when compressed, consistent with Frank’s findings that SUV_max_ decreased in the C7 segment of CSM patients [9].

Predicting the increase or decrease in SUV_max_ values based on MRI is an important basis for predicting surgical outcomes. Kenzo showed a negative correlation between the abnormal low intramedullary signal on T1-weighted images and the high intramedullary signal on T2-weighted images with a neurological improvement rate (*p* < 0.05) [11]. Furthermore, the SIR (Signal Intensity Ratio), indicating the ratio of increased lesion signal intensity to C7/T1 disc level signal intensity, demonstrated a negative correlation between neurological improvement rate and SIR on T1-weighted images (R = 0.617, *p* < 0.01), but its correlation with SIR on T2-weighted images was not significant (R = −0.256) [11]. However, in the radiomics model constructed in this study, we found a relationship between high T2 signal intensity and decreased SUV_max_. Additionally, in this study, through radiomics feature analysis, we discovered that features such as chaotic signals and smaller areas might also contribute to decreased SUV_max_.

### 4.3. Treatment Options for Patients with Cervical Spondylotic Myelopathy

Currently, there is still controversy over whether surgery should be actively performed on CSM patients with different severity degrees. Frank’s study suggests that in moderate to severe CSM patients, those exhibiting decreased 18FDG uptake did not show significant improvement postoperatively (pre-op mJOA = 11.6, post-op mJOA = 12.0, *p* > 0.05), while those exhibiting increased 18FDG uptake showed significant improvement (pre-op mJOA = 9.5, post-op mJOA = 13.6, *p* = 0.001) [10]. This indicates that for moderate to severe CSM patients, timely surgery can significantly improve symptoms before 18FDG uptake decreases. However, there are still questions regarding whether surgery should be actively performed for mild CSM patients. To explore the prognosis of mild CSM, the 24 patients enrolled in this study had relatively mild symptoms (median mJOA of 15 points). Follow-up results showed that for mild CSM patients, regardless of whether PET showed reduced uptake, active surgery could improve patient symptoms (SUV_max_ decrease group: pre-op mJOA = 15, post-op mJOA = 16, *p* = 0.012; normal SUV_max_ group: pre-op mJOA = 15.5, post-op mJOA = 17, *p* = 0.001). However, for patients without decreased SUV_max_, their symptom improvement was more significant (*p* = 0.043). Combined with the previous literature, we believe that patients with normal SUV_max_ can benefit from surgery regardless of the severity of the disease, although complete recovery is difficult for moderately severe patients. For patients with decreased SUV_max_ in mild CSM, symptoms can still improve after surgery, but for moderate to severe CSM patients, symptoms do not significantly improve.

**Figure 5:**
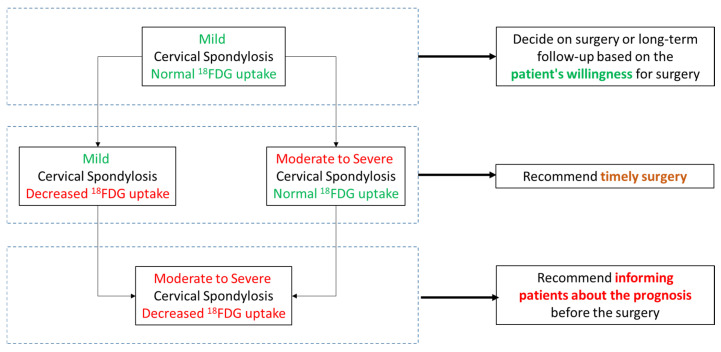
Proposed flowchart of the treatment of cervical spondylotic myelopathy.

Therefore, we built a treatment flowchart for CSM patients based on the MRI and PET findings, as Figure 5 shows. For mild CSM patients with normal SUV_max_, the decision to undergo surgery or long-term follow-up could be based on the patient’s willingness. For mild CSM patients with decreased SUV_max_ and moderate to severe CSM patients with normal SUV_max_, we recommended timely surgical treatment to prevent progression. For moderate to severe CSM patients with decreased SUV_max_, due to poor surgical outcomes, we recommend fully informing patients about the poor prognosis before surgery.

### 4.4. Other Methods for the Assessment of Cervical Spondylotic Myelopathy

Diffusion Tensor Imaging (DTI), an advanced neuroimaging technique for visualizing the integrity of white matter tracts, has gained considerable attention in the study of CSM in recent years. Studies have demonstrated that patients with mild CSM exhibit significantly higher fractional anisotropy (FA) values compared to those with moderate to severe disease, whereas the differences in FA between moderate and severe cases are not statistically significant [34]. Furthermore, DTI can assist in identifying the responsible segment of spinal cord compression based on localized FA reductions [35].

In terms of prognostic evaluation, dynamic DTI studies have shown that preoperative FA values measured in different cervical spine positions are significantly associated with postoperative functional recovery (*p* < 0.029) [36]. Beyond DTI, other multimodal approaches have also shown promise in the diagnosis and prognostication of CSM. For example, a deep learning model based on electroencephalography (EEG) achieved an accuracy of 92.5% in binary classification tasks for CSM diagnosis [37]. Additionally, intraoperative ultrasound monitoring has been found to correlate closely with JOA scores and may hold potential prognostic value [38]. In summary, these multimodal imaging and physiological signal analysis techniques offer novel, multidimensional approaches to the comprehensive evaluation of spinal cord function in CSM patients.

### 4.5. Limitations

This study has certain limitations. Firstly, the sample size is relatively small. As a prospective study, it included 24 patients with a total of 116 segments, with only 56 segments available in task two. Although this number surpasses or is comparable to previous related studies [9,10], for machine learning algorithms based on radiomics, further expansion of the sample size is necessary. Secondly, this study only analyzed T2 data, while prior studies have indicated that low signals on T1 also hold prognostic significance [11]. Therefore, future research will incorporate multimodal radiomics to construct machine learning models, thereby enhancing predictive accuracy. Thirdly, multi-center studies are needed to validate the stability and robustness of the model.

### 4.6. Future Directions

Future research should focus on expanding the cohort, particularly by including more moderate to severe CSM cases for model training and validation to enhance generalizability. In addition, combining radiomics with other biomarkers, such as physiological measurements or clinical assessments, may further improve the model’s predictive performance. These steps aim to advance toward a comprehensive decision-support system that can aid personalized treatment planning in CSM before surgery. The proposed method has the potential to preoperatively evaluate prognosis-related risk, especially in mild CSM patients, for whom surgical decision-making is often challenging.

## 5. Conclusions

This prospective study included a cohort of mild cervical spondylotic myelopathy (CSM) patients with follow-up assessments. Follow-up results indicated that the improvement in symptoms for the not-decreased SUV_max_ group was higher than that for the decreased SUV_max_ group. We recommend timely surgical treatment for mild cervical spondylosis patients with decreased SUV_max_ to prevent symptom worsening. Meanwhile, the machine learning algorithm based on radiomics completed two tasks: the automatic identification of compressed segments and the automatic identification of decreased 18FDG uptake segments. The results showed that the radiomics-based machine learning algorithm outperformed clinical doctors by 10% in predicting whether 18FDG uptake would decrease. This study also identified several imaging features related to decreased 18FDG uptake, including a smaller cross-sectional area, chaotic signals, and higher intramedullary signals, which provided an alternative to PET examinations. Radiomics-based prediction of SUV_max_ changes could potentially reflect spinal cord function and could be worth further exploration for its prognostic and clinical relevance.

## Figures and Tables

**Table 1:** Clinical characteristics of patients’ table.

	Decreased SUV_max_	Normal SUV_max_	*p*-Value
Num	10	14	
Age (years)	56.9 ± 11.4	57.9 ± 8.3	0.801
Gender (M, %)	7 (70%)	7 (50%)	0.349
Course (months)	42 (6–114)	12 (5.5–66)	0.465
BMI	26.2 ± 1.5	25.1 ± 3.0	0.284
Pre-op mJOA	15 (14–16)	15.5 (14–16)	0.732
Post-op mJOA	16 (15.75–17)	17 (16–17)	0.24
mJOA improvement	1 (1–1.25)	2 (1–2)	0.043

**Table 2:** Datasets of identifying compressed levels.

Task 1	Training Dataset					Test Dataset			
Level	Normal	Percent	Compressed	Percent		Normal	Percent	Compressed	Percent
Total	45	51.72%	42	48.28%		15	51.72%	14	48.28%
C2/3	16	100.00%	0	0.00%		5	100.00%	0	0.00%
C3/4	12	66.67%	6	33.33%		5	83.33%	1	16.67%
C4/5	4	22.22%	14	77.78%		2	33.33%	4	66.67%
C5/6	2	11.11%	16	88.89%		1	16.67%	5	83.33%
C6/7	11	64.71%	6	35.29%		2	33.33%	4	66.67%

**Table 3:** Datasets of identifying decreased ^18^F-FDG uptake levels.

Task 2	Training Dataset					Test Dataset			
Level	Normal	Percent	Decreased	Percent		Normal	Percent	Decreased	Percent
Total	32	76.19%	10	23.81%		8	57.14%	6	42.86%
C2/3	0	/	0	/		0	/	0	/
C3/4	6	100.00%	0	0.00%		1	100.00%	0	0.00%
C4/5	12	85.71%	2	14.29%		3	75.00%	1	25.00%
C5/6	10	62.50%	6	37.50%		2	40.00%	3	60.00%
C6/7	4	66.67%	2	33.33%		2	50.00%	2	50.00%

## Data Availability

The data and code used to support the findings of this study are available from the corresponding author on request.

## Abbreviations

CSM	Cervical spondylotic myelopathy
mJOA	Modified Japanese Orthopaedic Association scale
SHAPLEY	Shapley Additive exPlanations
LIME	Local Interpretable Model-Agnostic Explanations
SUV_max_	The maximum standardized uptake value
